# The height limit of a siphon

**DOI:** 10.1038/srep16790

**Published:** 2015-12-02

**Authors:** A. Boatwright, S. Hughes, J. Barry

**Affiliations:** 1 Department of Chemistry, University of Leicester, Leicester LE1 7RH, U.K; 2 Department of Chemistry, Physics and Mechanical Engineering, Queensland University of Technology (QUT), 2 George St, Brisbane, Queensland 4000, Australia

## Abstract

The maximum height of a siphon is generally assumed to be dependent on barometric pressure—about 10 m at sea level. This limit arises because the pressure in a siphon above the upper reservoir level is below the ambient pressure, and when the height of a siphon approaches 10 m, the pressure at the crown of the siphon falls below the vapour pressure of water causing water to boil breaking the column. After breaking, the columns on either side are supported by differential pressure between ambient and the low-pressure region at the top of the siphon. Here we report an experiment of a siphon operating at sea level at a height of 15 m, well above 10 m. Prior degassing of the water prevented cavitation. This experiment provides conclusive evidence that siphons operate through gravity and molecular cohesion.

Although the siphon has been used since ancient times, the means of operation has been a matter of controversy^1^,^2^,^3^,^4^,^5^,^6^. Two competing models have been put forward, one in which siphons are considered to operate through gravity and atmospheric pressure and another in which gravity and liquid cohesion are invoked. Key evidence for the atmospheric model is that the maximum height of a siphon is approximately equal to the height of a column of liquid that can be supported by the ambient barometric pressure. In this model, a siphon is considered to be two back-to-back barometers. Another piece of evidence in support of the atmospheric model is the fact that siphon flow can occur with an air bubble inside the tube so that there is no physical connection between the water molecules. Evidence in support of the gravity cohesion model is that siphons have been shown to operate under vacuum conditions^7^,^8^,^9^ and the model can explain a curious waterfall-like feature when a siphon is operating close to the barometric limit^10^.

Both siphon models–atmospheric and cohesion–predict that the maximum height of a siphon is dependent on the ambient barometric pressure. In the case of the atmospheric model, the pressure of the atmosphere is required to hold the column of water together. In the cohesion model, the limit is explained by the pressure at the top of the siphon falling below the vapour pressure of water, at the given temperature, so that cavitation occurs, i.e. the water starts to boil thereby breaking the column.

However, the cohesion model predicts that if cavitation can be prevented, the barometric height limit can be broken. The reason for the cohesion is that surfaces cost energy and the water/air surface is no different. For water, the surface energy is often referred to as surface tension. The surface energy of the water/air interface is 0.072 J/m^2^. It costs energy to make bubbles in water because of the energy of the bubble surface. For a bubble to be stable it must be supported either by internal pressure of a gas or by the equivalent tension (negative pressure) in the water. For gas in a bubble the pressure (*P*) is given by (1). This equation^11^ is exact for an ideal gas, but an approximation for a real gas.


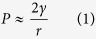


where *γ* is the surface energy (J/m^2^ or N/m), and *r* (m) is the bubble radius. A good benchmark pressure is the atmospheric pressure which is = 1.013 × 10^5^ Pa (N/m^2^). An internal pressure of one atmosphere (or equivalent tension in the water) could support a bubble of radius *r* where:


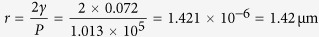


That is, an internal pressure of one atmosphere is generated by a bubble of 1.42 μm radius (a diameter of 2.8 μm). Equivalently, tension equal to the support of one atmosphere would occur for an empty bubble of diameter 2.8 μm. A smaller bubble would support greater water tension and a larger bubble a lesser water tension. A bubble of 2.8 nm diameter could tolerate water tension equal to 1000 atmospheres (100 MPa).

Many experiments have been performed to measure the tensile strength of water^12^,^13^,^14^,^15^,^16^,^17^,^18^,^19^,^20^and values as high as −150 MPa have been achieved^21^. All these experiments have been performed in static samples. In this paper we report, for the first time, a siphon operating at above the barometric limit at ambient atmospheric pressure. Thus we demonstrate the bulk flow of water under tension.

In an initial experiment, 60 ml of ordinary tap water with a 4 ml silicon oil-capping layer was held under a vacuum of <10^−3^ Pa for a period of more than three weeks. During the initial degassing process, significant volumes of gas were evolved from both the water and capping layers. This process is commonly attributed to boiling, but as qualified in subsequent sections, this effect is entirely due to dissolved gasses coming out of the water. A small amount of water (~2 ml) was evaporated from the initial volume, mainly due to the exposure of the surface of the water when large bubbles passed though the capping layer.

Once the water and capping layer were fully degassed, there was no further loss of either fluid. After allowing the vessel to return to atmospheric pressure for a short time, subsequent evacuations did not cause more gas to evolve from the water (video sequence 1). However, returning the container to the ambient air pressure for several hours did allow gas to be reabsorbed into the oil-capping layer, and over a longer period, into the water underneath. This gas was released again when the container was re-evacuated.

In the next experiment, the cohesive strength of water was tested using a simple inverted U-tube with the base exposed to vacuum, in the manner of a barometer (Fig. 1). Initially the U-tube was set to below the level of the surface of the liquid, while the glass vessel was evacuated, and all gases fully removed from above and within the liquids. When the partial pressure inside the vessel reduced to 7.5 ± 0.05 × 10^−1^ Pa the U-tube was raised by lifting the apex of the tube to a height of 300 mm above the surface of the oil. With a density marginally lower than that of water, the oil surface was assumed to be close to that of a hypothetical water vacuum interface. It was observed that the water formed a continuous column with no bubbles/cavities forming at the top of the tube (Fig. 2). The inverted U-tube was then held in this position for a period greater than four weeks. After this time, the U-tube was tilted further, so the apex was 400 mm above the surface, while reducing the partial pressure above the liquid to 5 ± 0.05 × 10^−3^ Pa. In this position the water column was observed to be stable with no bubbles seen to evolve in the U-tube even after several hours.

To test the ability of water to maintain cohesion under conditions of flow, a glass siphon was constructed such that both reservoirs could be held under high vacuum (Fig. 3), in a manner similar to that performed previously by Noaks^8^. In this arrangement, during the degassing process with the U-tube set below the oil, the level of the liquid in both reservoirs was equal with half filling each. When the U-tube was then raised to a vertical position, an offset in position allowed one reservoir to rise further than the other leading to a small height difference. With the U-tube initially in the lower position, water was degassed to a partial pressure of 9.5 ± 0.05 × 10^−1^ Pa. The apex of the U-tube was the raised 300 mm and water observed to flow from the higher chamber to the lower *via* the siphon tube into the lower chamber (video sequence 2).

While the flow was initiated independently of atmospheric pressure within the siphon, it was noted that the movement of the reservoirs between the static and flowing conditions exposed surfaces that were previously covered with water. As this happened the pressure in the vacuum region was observed to rise above 10^3^ Pa. Realising that this represented a fundamental flaw, in this, and in previous attempts by others at producing a water siphon under vacuum conditions, it was deemed that a moderate length siphon could not conclusively discount the effects of vapour pressure on supporting the column.

In order to discount the effect of external pressure acting on the liquid column, a second siphon was constructed, operating under atmospheric conditions, with a height above the nominal barometric limit of 10 m, using water degassed using a vacuum desiccator (Fig. 4).

The siphon height, defined as the vertical distance between the surface of the water in the upper reservoir and apex of the tube, started at 1498 ± 2 cm and increased to 1504 ± 2 cm (Fig. 5). The barometric pressure during the experiment was 99.8 ± 0.1 kPa. The experiment was repeated a number of times and an example is shown in the relevant supplementary video (video sequence 3). After opening both taps at the base of the pre-primed siphon, water was observed to flow out of only the lower of the two siphon legs (video sequence 4). Approximately 400 ml of water flowed from the upper to lower reservoir in 850 s corresponding to a flow of 4.7 ± 0.05 × 10^−7^ m^3^ s^−1^ and average velocity of 1.7 ± 0.05 × 10^−2^ m s^−1^.

To measure the effects of capillary action in making any contribution to lifting the water within the siphon tube, one end of the empty siphon tube was immersed in the degassed water, which was open to air, while the other open end of the tube was held above the level of the liquid. As no differential was observed between the heights of the liquid inside the nylon tube and outside, capillary action was discounted as playing any significant role in the siphon process.

The ability to completely degas water has always represented a significant challenge in performing experiments investigating liquid tensile strength. It is widely known that the great variance observed both within and across different methods investigating the properties of water is due to the unpredictable nature of the gases dissolved within^22^. In water free of all dissolved gases, bubbles only form when the energy gained in forming a cavity is greater than the binding energy of the surrounding molecules.

Cavity formation in fully degassed water thus represents the limit of cohesion of the water molecules. Of the methods used, such as boiling, sonication, membrane degasification and freeze pump thaw, those where water is exposed to a vacuum are generally considered to be the most effective at removing all dissolved gases. This can be understood by extrapolating to the limit of Henry’s law


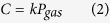


where C is the solubility of a gas at a fixed temperature in a particular solvent, *k* is Henry’s constant and *P*_*gas*_ the partial pressure of the gas above the liquid. Accordingly, at zero pressure the amount of dissolved gas should also equally be zero. However, due to practical constraints it is difficult to achieve pressures above the surface much below that of the vapour pressure, which for water at 20 °C is approximately 2.33 kPa, and consequently some dissolved gases will always be present.

At temperatures above freezing, and below the boiling point, the bonds between adjacent water molecules at the liquid air interface are continuously being broken and reformed. This constant exchange between molecules leaving and re-joining is generally at equilibrium at atmospheric pressure and room temperature, which is why we see liquid water so abundantly on earth. However, once the pressure above the interface is reduced, or the temperature of the liquid below increased, the equilibrium shifts and water molecules are on average lost from the bulk liquid.

A simple method to overcome water loss is to change the energy barrier at the surface of the water by applying a layer of immiscible liquid above the surface. By floating a liquid with low specific gravity and ultra-low vapour pressure over the water, molecules at the interface are unable to leave the water and migrate through the capping liquid to the surface. Thus evaporative loss that normally occurs below the water vapour pressure is considerably reduced, if not entirely negated.

After initially degassing the water, there was no further evaporative loss or cavitation within the bulk liquid, or at any interface when the ambient pressure was below 10^−3^ Pa. While it could be argued that the oil was applying a downward force on the water raising the pressure above that of the vapour point, with a capping layer of only 5 mm, the oil would contribute a downward pressure of less than 43 Pa.

It was also observed that with the surface of the water capped by oil during the degassing stage there was only a drop in temperature, measured on a mercury thermometer, when the water surface became exposed to the vacuum, as happened when large bubbles exploded at the surface. The temperature of the water would then gradually increase over time returning to the ambient temperature of the lab. This very slow temperature increase was attributed in part due to some radiant energy through the Perspex front of the chamber but predominantly from thermal conduction through the apparatus. Over the period of 3 weeks, when under vacuum, the temperature of the water was observed to remain steady at approximately 21 °C.

This surprising behaviour is explained by considering the dynamics of evaporation, where on average the most energetic molecules tend to leave the surface first. In this case, by increasing the energy barrier at the surface, no evaporation can occur, therefore there is little or no net loss of energy from the system leaving the temperature constant. Consequently, while the oil acts as an effective barrier to the evaporative loss of water, it does not prevent gas transport in either direction, or significantly change the pressure gradient within the liquid. Consequently these experiments show that while exposed water does evaporate under low partial pressures, as would be expected, internal cavitation or nucleated boiling does not occur at room temperature even under extremely low ambient pressures.

For a siphon with dissolved gases the maximum height (*h*_*m*_) of a siphon is


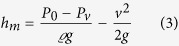


where *P*_*0*_ is the ambient atmospheric pressure, *P*_*v*_ is the vapour pressure of water, *v* the mean velocity of the water and the other symbols are as previously defined in this paper. The expression for the atmospheric model is the same as equation (3) except with no *P*_*v*_ term.

The siphon in the experiment described in this paper was clearly operating above the barometric limit, which, at the given barometric pressure was 10.18 ± 0.01 m for the atmospheric model and 9.94 ± 0.01 m for the cohesion model (ignoring the negligible velocity term). Therefore, it is evident that atmospheric pressure plays no part in carrying the water over the apex of the siphon tube. Therefore it is clear that a new equation for the maximum height of a siphon is required for situations where cavitation does not occur.

The new equation is much simpler and is


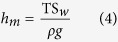


where TS_w_ is the tensile strength of water. So for example if the tensile strength of a sample of water was 1 MPa, the maximum height of a siphon would be about 100 m. In the case of the siphon is this experiment we can say that the tensile strength of the water was greater than −0.15 MPa.

Extrapolating these results from even the most conservative experimental measurements of the tension under which cavitation occurs it is possible that the cohesive strength of fully degassed water is able to support a continuous vertical column greater than several hundred meters. While the experiment performed here did not reach anywhere near the absolute limit predicted it does shed light on the stability of flowing water under tensile stress and the possibility of constructing apparatus of suitable dimensions to test such a limit. These experiments also lend support to the cohesion-tension theory of sap ascent in trees. It would be interesting to perform further experiments to see if it is possible to operate a flowing siphon at above 100 m. If tensions as high as the transient tension of several 100 bar can be maintained at the apex of a siphon, then in principle a siphon should work up to a height of several km. However, it would be challenging to verify this experimentally, requiring a helicopter or UAV with a ceiling of several km capable of supporting several kg of water-filled tubing and cable supporting the siphon. It would also be interesting to repeat the experiment with a larger diameter tube. In view of the many anomalies of bulk water^23^, it would be interesting to explore the physical properties of water in the negative pressure regime of a siphon above 10 m.

## Methods Summary

In previous attempts to degas water in vessels constructed from materials such as metal and rubber it was observed that the surfaces acted as effective nucleating sites leading to continual bubble formation and water loss. Consequently, in the experiments described here, glass or nylon was used for surfaces that came into contact with the water. To aid observation, a red water-soluble dye was used (Rhodamine 640 Perchlorate, Exciton Inc), after first performing each experiment using untreated tap water. There was no noticeable difference in nucleation/degassing rates observed or absolute pressure attained between the ordinary tap water and the dyed water throughout all subsequent experiments. As the dye was not soluble in oil the capping layer remained transparent and colourless throughout.

**Figure 1:**
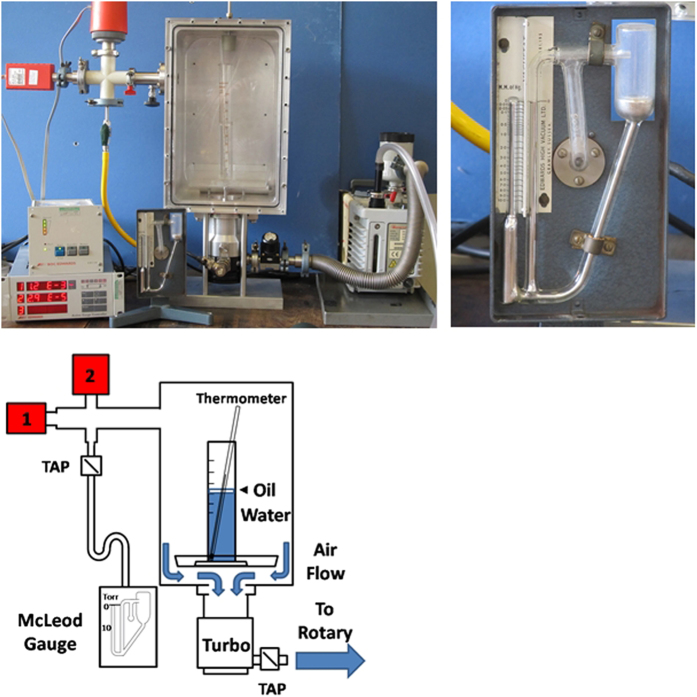
Upper picture: Experimental apparatus for the degasification of water; Right picture: Expanded view of McLeod Gauge; Lower diagram: The 100 ml graduated glass measuring cylinder is filled with 60 ml of water and capped with 5 ml of oil, which stands on a small Perspex tray above the turbomolecular pump. Pressure gauges are marked 1) APG-M-NW16, 2) AIM-S-NW25 and McLeod.

For the initial experiment, vacuum pressure measurements were made using three independent gauges mounted on the side of the Perspex fronted vacuum vessel (Fig. 1). The vacuum gauges were located on the far side of the chamber such that the pressure readings were independent of any molecular flow between the oil/water surface and the vacuum pump, thus measuring the static pressure in the chamber. The three gauges consist of an Edwards Active Priani Gauge (APG-M-NW16), Edwards Active Inverted Magnetron Guage (AIM-S-NW25) and Edwards Vacustat McLeod gauge, with operating ranges of atmosphere to 1 × 10^−2^ Pa, 1 to 1 × 10^−6^ Pa and 10 to 0.01 mm of Hg for each respectively. To provide a complete pressure range once the pressure in the chamber was reduced below the operational limit of the Pirani gauge the AIM gauge was then used to record the pressure. To provide independent verification of the two electronic gauges a mercury-filled Edwards Vacustat McLeod gauge was also attached to the chamber. While less accurate than the two electronic gauges, the McLeod gauge was able to show that the vacuum was of a similar order of magnitude and that the electronic gauges were not unduly affected by water in the partial atmosphere.

In the first degassing experiment, 60 ml of ordinary tap water was held in a 100 ml measuring cylinder placed inside a Perspex fronted vacuum chamber (Fig. 1). To measure changes in temperature during the evacuation process a calibrated (−10 to 100 °C) glass mercury filled thermometer was placed into the water inside the measuring cylinder. A 5 mm thick layer of Satorrlene Normal (Duravac Products Ltd) diffusion pump fluid (with a saturated vapour pressure 3.2 × 10^−6^ Pa at 25 °C, specific gravity 0.863 g/cm^3^ at 25 °C, viscosity 60 mPa s at 40 °C) was then floated above water. As the two liquids are immiscible the less dense silicon oil formed a visible capping layer over the surface of the water. Once the water was completely covered with a layer of oil the air was removed from the chamber by evacuating through a 6 cm diameter hole in the base of the chamber to which a BOC Edwards EXT70 turbomolecular turbo vacuum pump (B722–01–000) pump is directly attached. Starting at atmospheric pressures this was achieved by opening the SP16K Edwards speedy valve at the base of turbo pump which was connected, *via* a 60 cm long KF25 vacuum bellows, to an Edwards 5 rotary vane pump (A653–01–903). Once vacuum pressures below 1 Pa were achieved the turbo molecular pump was then switched on reducing the pressure to below 1 × 10^−3^ Pa.

As the volume above the fluids was evacuated to pressures below 3 kPa, dissolved gasses were immediately released at a very high rate, appearing in a manner not unlike that of nucleated boiling. After the initial burst of gas the rate of release slowed significantly with occasional bubbles still appearing after a period of many hours. At intermediate stages in the degassing process the low partial pressure caused occasional bubbles to expand explosively through the surface of the oil, exposing the water to the vacuum. When this occurred a small amount of water at the surface would evaporate leading to further bubbles in a continuous cycle, leading to a marked drop in temperature observed on the thermometer. To prevent complete evaporation of the sample, and possible freezing of the water, the vacuum vessel was immediately isolated until the partial pressure rose and bubble formation abated. Once the water surface had settled and a continuous layer of oil reformed over the water pumping was resumed.

During later stages of the degassing process it was necessary to slow or temporarily halt the vacuum pump to allow the layer of oil to reform and gas emerge, as the flow of small bubbles through the oil had turned it into translucent foam. The pump-stop-pump process was then repeated until the oil became fully transparent. When no more gas was observed to evolve from either liquid the partial pressure was then reduced to <10^−3^ Pa by switching on the turbo pump.

For the inverted U-tube and vacuum siphon experiment, pressures were measured using an Edwards Priani gauge mounted at the top of a short 400 mm long, 6 mm inner diameter, flexible reinforced plastic hose, in front of two rubber sealed vacuum taps connecting to the vacuum pumps and to air. Pumping of the vacuum was achieved using an Edwards diffusion pump, roughed using an oil-filled rotary vacuum pump (Edwards 8). When not connected to a sample chamber, a partial pressure of less than 10^−4^ Pa was recorded using an Edwards Penning gauge upstream of the diffusion pump. During initial degassing of the samples the vacuum line could be diverted directly to the rotary pump. The rotary pump was also used when the sample was pumped continuously over extended time frames.

The U-tube glass vessel was made by connecting a 500 mm long, 6 mm inner diameter glass U-tube to the glass reservoir. The base of the U-tube was shaped such that as the apex was raised vertically the connections to the reservoirs would remain below the surface of the liquid. During the degassing process the U-tube was angled downwards away from the surface of the evacuated volume, allowing bubbles formed in the U-tube to float freely to the surface of the water before passing through the oil. It was observed that at later stages of the degassing process, bubbles formed within the U-tube would expand, almost filling the entire length of the tube. Once the bubble reached the surface, the water columns would re-join at great speed, causing a loud ringing noise within the glass vessel, often nucleating new bubbles within the column. Once the degassing process was complete after a period of several hours, the apex of the U-tube was then raised while the volume above the liquids was continually evacuated using the Edwards rotary pump and then at reduced partial pressures using a vacuum diffusion pump. A similar construction and degassing method was used for the vacuum siphon. These methods were then repeated for each of the U-tube and vacuum siphon experiments with dye added to the water.

To create the siphon above the barometric limit, a 30 m long 6 mm inner diameter flexible nylon tube (RS components) was used. Attached at either end of the tube were two stainless steel vacuum taps. Prior to filling with the previously degassed water, the tube was continually flushed with tap water for 4 hours to remove any deposits from within the pipe. The tube was then connected at one end to the vacuum pumps and evacuated continually for a period of 48 hours to allow all volatile compounds to be removed. Priming of the tube was achieved by placing the closed end of the evacuated tube into the degassed water, which was then opened allowing the water to flow up the tube, while the other end remained open to the vacuum system. Care was taken to prevent the capping oil from entering the tube during this process. Once the tube was entirely filled with degassed water, both ends of the tube were closed ready for the siphon to be set into position.

Before setting the tube in position the siphon was first inverted so that the ends of the tube were at the highest point with the bend at the lowest. This was done to allow additional degassed water to be added as the increased weight caused a slight expansion in the length of the tube. Once the extra water was added the tube was re-inverted with the bend at the apex and the legs hanging straight down into the reservoirs. To prevent kinks occurring in the hose at the apex the siphon the tube was set into a pulley of 12 cm diameter. Once primed, one end of the siphon was set into a reservoir containing more degassed water, with the other venting 30 cm lower inside an empty 1 litre glass beaker with both reservoirs open to the air. Taps at both ends of the tube were then opened so that the liquid could flow freely over the 14.5 m rise into the lower reservoir (video sequence 4). Once the upper reservoir was nearly depleted of liquid, the end was lifted out of the liquid allowing air to flow into the base of the tube.

Throughout the siphoning process no bubbles were observed in the tube, however small bubbles were observed to emanate from the lower end of the siphon during in the final stages of draining the tube. These bubbles were thought to come from air trapped in the tap and the glass vessel at the base of the tube, which became dislodged by the fast flowing liquid.

## Additional Information

**How to cite this article**: Boatwright, A. *et al.* The height limit of a siphon. *Sci. Rep.* **5**, 16790; doi: 10.1038/srep16790 (2015).

## Supplementary Material

Supplementary legends

Video 1

Video 2

Video 3

Video 4

## Figures and Tables

**Figure 2:**
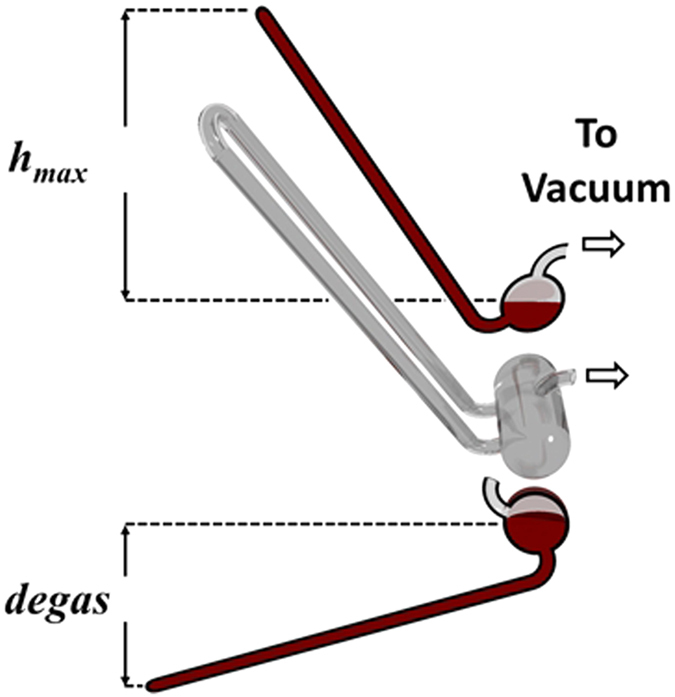
Diagram of a water-filled U-tube barometer. The lower figure shows the position during evacuation and degassing of the water with an oil-capping layer and the upper figure shows the U-tube tilted into position while the base is held under vacuum.

**Figure 3:**
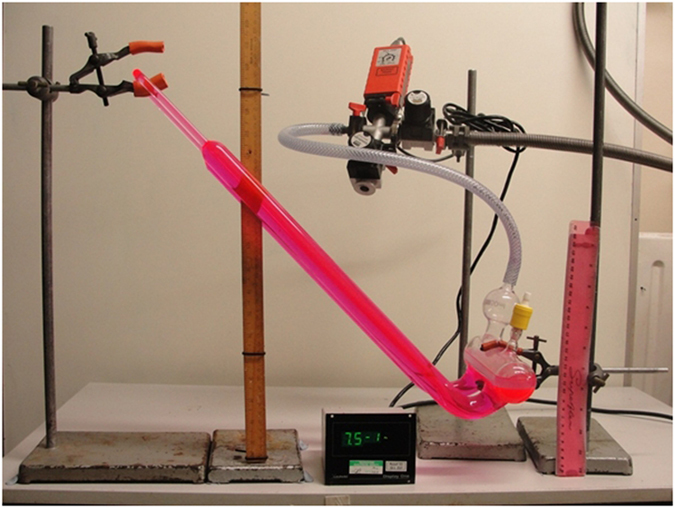
Photograph of U-tube barometer while under vacuum. Pressure readings are in Pa and the height of the apex is 300 mm above the surface of the liquid.

**Figure 4:**
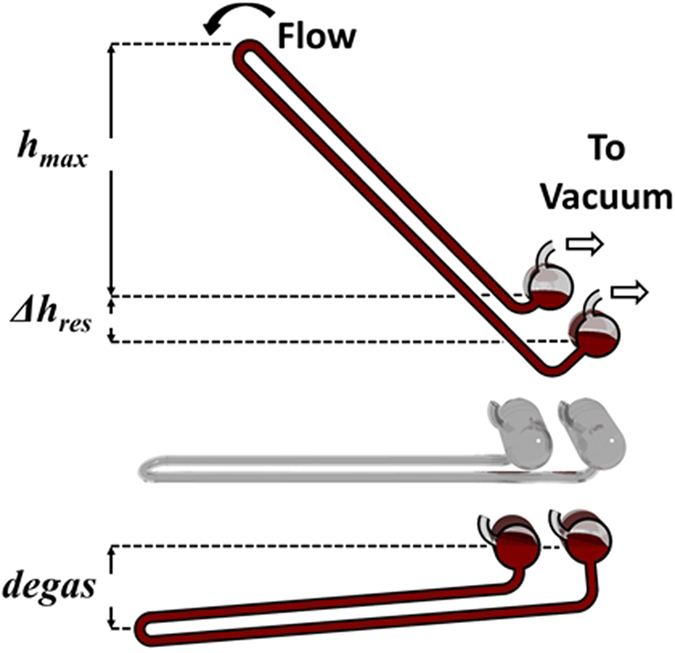
Diagram of a water siphon under vacuum. The lower figure shows the position during evacuation and degassing of the water with oil capping layer and the upper figure shows the position of the siphon tilted with liquid flowing from the upper to the lower reservoir, while each reservoir is held under vacuum.

**Figure 5:**
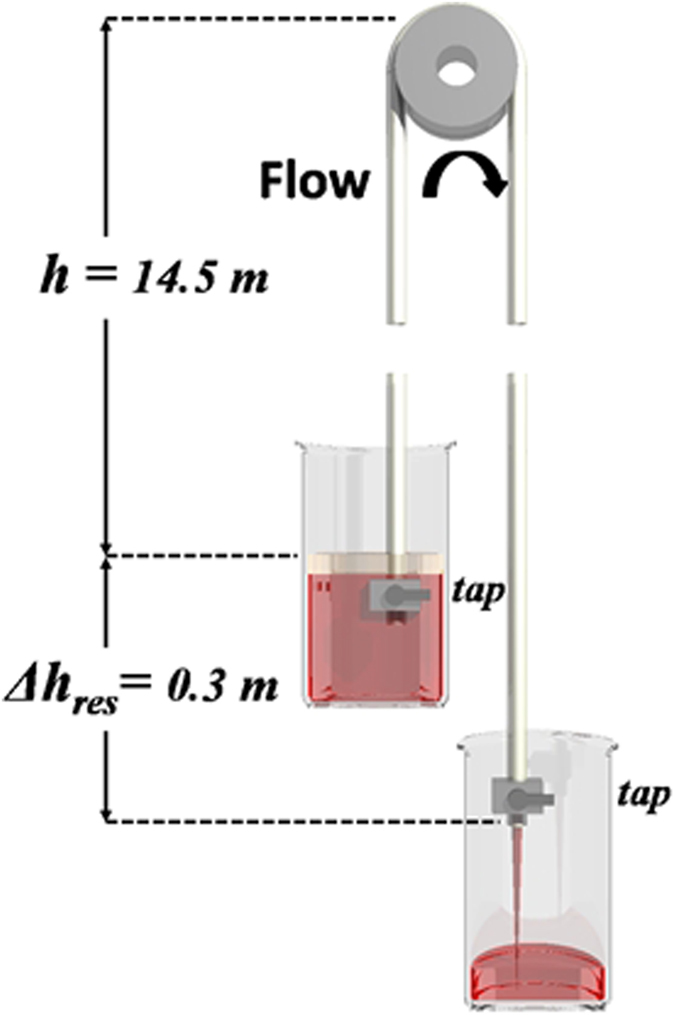
Diagram of a siphon taller than the barometric limit with the reservoirs open to air. Water in the upper reservoir is capped with a 5 mm layer of silicon oil. A pulley is used at the apex to support the length of tube and prevent kinks in the pipe.
