# Corrigendum: The height limit of a siphon

**DOI:** 10.1038/srep46792

**Published:** 2017-05-02

**Authors:** A. Boatwright, S. Hughes, J. Barry

Scientific Reports 5: Article number: 1679010.1038/srep16790; published online: 12 02 2015; updated: 05 02 2017

We wish to acknowledge a related 1995 experiment performed by Andrew K Fletcher (http://inclinedbedtherapy.com), in which a 48 m length of 6 mm bore tubing, filled with degassed water was lifted 24 m vertical against Brixham Cliffs. A 50 ml bolus of coloured saline solution in the middle of the 48 m tubing was introduced prior to the tubing being raised to 24 metres vertical by its centre as an inverted U with each open end in a vessel containing degassed water, which caused water to flow from one container to another. The experiment showed that water may be raised above the assumed 10 meter limit in an open-ended tube. The two containers were at the same height, as were the ends of the tubing. This circulation observed was not a ‘classic’ siphon where water flows between two reservoirs at different heights. In this experiment, flow was driven by the difference in density between saline and fresh water. Fletcher used the 24 m experiment to support a new theory of how sap circulates in trees.

In our article, we determined that a siphon could operate above 10.3 m using an experimental design with some similarities to Fletcher’s experiment.

