# Supplementary material for: The height limit of a siphon
Source: Sci Rep. 2015 Dec 2;5:16790. doi: 10.1038/srep16790 (PMC4667279; doi:10.1038/srep16790)
Supplement: Supplementary legends [file srep16790-s1.pdf]

The height limit of a siphon

A Boatwright[1], S Hughes[2], J Barry[2]

Video 1. When a lid is momentarily removed off a flask containing degassed water, replaced and vacuum re applied no more gas evolves from the water.

Video 2. When a glass siphon containing degassed water is raised, water flows from one bulb to another. When the siphon is lowered the water flows in reverse.

Video 3. A 15 m high siphon in a stair well, ready to flow.

Video 4. Degassed water with an oil-capping layer flowing in a 15 m high siphon.
